# Investigating the expression pattern of the *OsAPx1 *gene promoter in rice

**DOI:** 10.1186/1753-6561-8-S4-P92

**Published:** 2014-10-01

**Authors:** Julio Garighan, Carolina Ribeiro, Márcia Margis-Pinheiro

**Affiliations:** 1 Universidade Federal do Rio Grande do Sul, Porto Alegre, Brazil

## Background

Ascorbate peroxidase (APx) is a key enzyme of the antioxidant metabolism, catalyzing the decomposition of hydrogen peroxide (H_2_O_2_) in water, using ascorbate as an electron donor. The H_2_O_2 _is a reactive oxygen species (ROS) produced constantly by aerobic metabolism. Under biotic and abiotic stress the level of H_2_O_2 _increases and, in large quantities, can cause cellular damage. In rice, there are eight APx genes that encode products target to different subcellular compartments: cytosol, peroxisoma, mitochondria and chloroplast. *OsAPx1 *gene encodes a cytosolic isoform of APx. The study of promoters is an important tool that allows to analyze the overall expression pattern of genes in plants.

## Methods

A sequence of approximately 2kb preceding the translation initiation site of the *OsAPx1 *gene was isolated, cloned into pENTR vector and recombined in pHGWFS7 vector, whichallows the fusion of the promoter sequence with two report genes, *Gfp *and *Gus*, and confers resistence to hygromycin. The construction was named pPROM1. The transformation of rice calli, originated from *nipponbare *cultivar seeds, was performed via *Agrobacterium tumefaciens*. The transformed calli were grown in selection medium with hygromycin, regenerated into plants, acclimatized in a greenhouse and the confirmation of transgene was verified by PCR using specific primers for the *Hpt *and *Gus *genes. For visualization of expression pattern of the promoter, by GUS histochemical assay, samples of plants were collected and analyzed by *X-gluc *histochemical assays. The segments were incubated in 1 mM*X-gluc *solution at 37°C for 16h. After reaction, green tissues were incubated in 70% ethanol for chlorophyll discoloration. In the *in silico *analysis of cis-elements in the promoter region of *OsAPx1 *was used the following databases available online:-PlantPan (http://plantpan.mbc.nctu.edu.tw/) and PlantCare (http://bioinformatics.psb.ugent.be/webtools/plantcare/html/)

## Results and conclusions

Nine lines of transgenic plants expressing *Gus *under the control of the *OsAPx1 *promoter were obtained. The GUSexpression was observed in leaf (especially in leaf mesophyll), ligule and in wounded regions. These results show that *OsAPx1 *gene seems to be expressed in green tissues and to respond to damage. Apparently, there is no change in the expression pattern during different development stages. The *in silico *analysis demonstrates the presence cis-elements responsive to hormones, drought and light.
